# Highlights and recent developments in skin allergy and related diseases in EAACI journals (2018)

**DOI:** 10.1186/s13601-019-0299-y

**Published:** 2019-11-20

**Authors:** C. A. Akdis, J. Bousquet, C. E. Grattan, P. A. Eigenmann, K. Hoffmann-Sommergruber, I. Agache, M. Jutel

**Affiliations:** 1 Swiss Institute of Allergy and Asthma Research (SIAF), University Zurich, Davos, Switzerland; 2 MACVIA-France, Fondation Partenariale FMC VIA-LR, CHU Arnaud de Villeneuve, 371 Avenue du Doyen Gaston Giraud, 34295 Montpellier Cedex 5, France; 3 INSERM U 1168, VIMA: Ageing and Chronic Diseases Epidemiological and Public Health Approaches, Villejuif, Université Versailles St-Quentin-en-Yvelines, UMR-S 1168, Montigny le Bretonneux, France; 4 Charité, Universitätsmedizin Berlin, Humboldt-Universität zu, Berlin, Germany; 5 Department of Dermatology and Allergy, Comprehensive Allergy Center, Berlin Institute of Health, Berlin, Germany; 6 St John’s Institute of Dermatology, Guy’s Hospital, London, UK; 7 Pediatric Allergy Unit, University Hospitals of Geneva, Geneva, Switzerland; 8 Department of Pathophysiology and Allergy Research, Medical University of Vienna, Vienna, Austria; 9 Transylvania University Brasov, Brasov, Romania; 10 ALL-MED Medical Research Institute, Wroclaw, Poland

**Keywords:** Atopic dermatitis, Urticaria, EAACI

## Abstract

The European Academy of Allergy and Clinical Immunology (EAACI) supports three journals: Allergy, Paediatric Allergy and Immunology as well as Clinical and Translational Allergy. The major goals of EAACI include (i) supporting health promotion in which the prevention of allergy and asthma plays a critical role and (ii) disseminating the knowledge of allergy to all stakeholders including the EAACI junior members. Substantial progress was made in 2018 in the identification of basic mechanisms of atopic dermatitis and urticaria and the translation of these mechanisms into clinics. Many large epidemiologic studies and meta-analyses have been the highlights of the last year.

## Introduction

The European Academy of Allergy and Clinical Immunology (EAACI) supports three official journals: Allergy, Paediatric Allergy and Immunology as well as Clinical and Translational Allergy. The major goals of EAACI include (i) supporting health promotion in which the prevention and control of allergy plays a critical role and (ii) disseminating the knowledge of allergy to all stakeholders including the EAACI Junior Members. The EAACI journals reported advances in allergy in 2017 [1, 2] and 2018 [3]. This paper summarizes the achievements of 2018 in atopic dermatitis and urticaria. The position papers and EAACI/WAO/GA^2^LEN guidelines are summarized.

## Atopic dermatitis

### Mechanisms

Atopic dermatitis (AD), commonly known as eczema, is a chronic skin disorder associated with skin barrier dysfunction that is characterized by dry, itchy skin (pruritus). Interleukin-31 (IL-31) secreted by T-helper 2 (T_H_2) cells induces the itchy symptoms. The role of IL-31 in the pathogenesis of AD and novel therapeutic strategies targeting its receptor have been recently reviewed [4].

AD patients have an altered skin microbiome composition characterized by an increased colonization of *Staphylococcus aureus* (*S. aureus*) which is associated with disease severity. In addition, AD patients have a reduced expression of toll-like receptor-2 (TLR2) receptors in Langerhans cells (LC) and inflammatory dendritic epidermal cells (IDEC) compared to healthy controls [5]. Ex vivo human skin models were treated with the TLR2 ligand Pam3Cys, a mimic of *S. aureus*. In contrast to healthy skin, LC and IDEC lacked maturation and had a strong spontaneous migratory activity. The supernatant of AD skin showed significantly reduced levels of IL-6 and IL-10 and an overexpression of IL-18.

A novel mechanism involved in epidermal barrier dysfunction was recently reported in a mouse model of allergic inflammation and in AD patients [6]. It was found that IL-24 downregulates filaggrin expression and is produced in keratinocytes by the stimulation of IL-13 in a periostin-dependent manner. Elevated levels of IL-24 and activated STAT3 were found in the epidermis of the mouse model and of AD patients.

The role of the pruritogenic mediator endothelin-1 (ET-1) in the pathogenesis of AD was recently investigated in mouse bone marrow-derived dendritic cells (BMDCs) [7]. ET-1 was significantly upregulated in the epidermis of AD patients and stimulated the production of Th1 and Th17 cytokines from BMDCs in a concentration-dependent manner. This switch to a Th17/Th1 response may contribute to the chronic lesions in AD patients.

Studies in a human AD-like mouse model revealed novel mechanistic insights that support a link between cell infiltration of the hypodermis to local mast cell activation and elevated levels of sphingosine-1-phosphate (S1P) after antigen exposure [8]. Neuromedin U (NMU) released from keratinocytes induces the degranulation of mast cells via pertussis toxin-sensitive G protein-coupled receptors [9].

Exposure to air pollution aggravates the symptoms of AD in children in the population studies. Variability in the individual patient’s response from individual susceptibility needs to be explored. In 89 children aged 0–6 years with AD, daily manifestation of symptoms was recorded for an average of 257 days and confronted to pollution levels [10]. In the overall analysis, ozone, particulate matter < 10 μm (PM_10_), NO_2_, SO_2_, and CO had a significantly positive association, whereas temperature and relative humidity were negatively associated with AD symptoms. However, responses of AD children to air pollution and weather variables were inconsistent among individuals.

### Epidemiologic studies

Earlier studies have shown an association between the month of birth and the severity of atopic dermatitis. In one study, Armenian children were investigated for the severity of their eczema in relation to the month of birth. It was found that children born in the winter had more severe eczema. The authors hypothesized, in relation to earlier findings, that the severity of eczema could be related to an early exposure to common environmental allergens [11].

Data of the epidemiology of atopic dermatitis were collected in an online survey from patients in the US, Canada, France Germany, Italy, Spain, UK, and Japan [12]. The respondents were characterized and selected if they met the UK Working Party/ISAAC criteria and had been previously diagnosed by a physician. There was a decreased prevalence of AD with age and, except for the US, a higher prevalence among women. Only a small portion suffered severe AD across all regions. The severity of AD varied according to the different assessment scales used (Patient-Oriented SCORAD, Patient-Orientated Eczema Measure, and Patient Global Assessment).

The plan for the Preventing Atopic Dermatitis and ALLergies (PreventADALL) in children study has been recently reported [13]. Its main objectives are the prevention of allergic diseases by assessing early-life risk factors, including microbial diversity, diet, lifestyle, and gene–environment interactions, using simple cost-efficient strategies. Children born by cesarean section and by assisted birth are at an increased risk of developing flexural eczema in childhood/early adulthood [14].

There are limited and conflicting reports on the long-term clinical course of AD. A systematic review and meta-analysis of 2080 references and 7 birth cohort studies with a total of 13,515 participants indicated that there is a similar prevalence of AD in childhood and adolescence [15]. Undergoing thymectomy in early childhood was associated with a reduced risk of AD but was only statistically significant in a model where time-dependent changes were not included [16].

### Severity scores

Reliability and validity of the AD Symptom Score (ADSS) were studied in 307 children and adolescents with AD [17]. Parents or caregivers were asked to record daily symptoms of the patients (itching, sleep disturbance, erythema, dryness, oozing, and edema) using a scale of 0–4. The ADSS was found be a useful tool for the self-assessment of skin symptoms in children with AD.

A recent study has validated the use of patient-reported AD severity assessment using a single question with a simple scoring system of mild, moderate and severe [18]. The responses from 265 adult patients significantly correlated with other outcome measures, including oSCORAD, SCORAD, EASI, BSA, NRS-itch, POEM, and DLQI, and were further confirmed by a dermatologist.

### Multimorbidities

The association between AD and cardio-metabolic risk factors is not fully understood, partly due to the lack of validated questionnaire-based methods to identify adults with AD. In an attempt to investigate the association of cardio-metabolic risk factors in AD patients, data collected from 9656 Danish adults were analyzed using three different cardiovascular risk questionnaires [19]. There was a large discrepancy in the results from each questionnaire and so a definite conclusion could not be reached. These results highlight the need for clinical diagnosis of AD by a dermatologist and the fact that responses from questionnaires should be carefully interpreted.

In a nationwide, population-based cohort study (Taiwan’s National Health Insurance Research Database), the association between obstructive sleep apnoea (OSA) and AD was sought in 120,736 children [20]. This study revealed an increased risk of obstructive sleep apnoea in children with AD. Therefore, comprehensive evaluation and aggressive risk reduction for obstructive sleep apnoea are recommended in these patients.

AD can significantly impact quality-of-life to the point that it can affect mental health. Data collected from the Danish health registry and a population-based questionnaire indicated that AD patients had an increased risk of mild anxiety, depression and suicidal ideation compared to non-AD subjects but did not result in psychiatric hospitalization or suicide [21]. Early antihistamine exposure for the treatment of AD was associated with increased attention-deficit/hyperactivity disorder symptoms in children aged 6–12 years. The study questionnaire asked parents whether their child had used systemic antihistamines to treat AD but did not distinguish between sedating and non-sedating H1-antihistamines. The authors noted that there is a need to further investigate the role of sleeping problems and its treatment with (sedating) antihistamines in young children concerning early-life development and the potential risk for ADHD in children with AD [22].

### Prevention and treatment

The protective effect of the Bacillus Calmette–Guerin (BCG) vaccination to reduce the risk of allergic diseases, including AD, is unclear. A recent study from the Danish Calmette (2012–2015) found that the clinical outcome of the BCG vaccination differed for children with and without atopic predisposition [23]. The cumulative incidence of AD was reduced by 16% in infants with an atopic predisposition. Oral antigen administration in mice has a protective effect against AD by promoting the increased expression of genes involved in the regulation of Th2 inflammatory responses and skin barrier function [24].

A randomized, double-blinded, placebo-controlled trial assessed the effects of melatonin administration on disease severity and sleep quality in 70 children with AD [25]. Overall, melatonin supplementation had beneficial effects on disease severity, serum total IgE levels, and on the Children’s Sleep Habits Questionnaire (CSHQ).

The efficacy of allergen-specific subcutaneous immunotherapy (SIT) as a curative treatment for atopic dermatitis remains controversial. A murine model was established to investigate the clinical efficacy of SIT [26]. The DfE-treated NC/Nga mice showed clinical, histological and immunological improvement with elevated levels of IL-10 producing Treg cells and NK cells.

The potential use of superoxide dismutase 3-transducer (SOD3) mesenchymal stem cells (MSCs), as a novel cell-based therapy for AD, was demonstrated in a mouse model of OVA-induced AD-like skin inflammation [27]. Mice with AD that received a subcutaneous administration of SOD3-MSCs showed an improvement of skin thickening and inflammation compared to control mice. The reduced skin inflammation was attributed to the inhibition of the histamine H_4_ receptor, MAPK/NFkB activation and JAK/STAT signalling.

Maternal exposure to a farming environment protects newborns against allergic diseases including AD by modulating the neonatal TLR-Tregs-Th axis [28].

In cats and dogs, there are pathogenetic similarities with human AD. This is often a difficult disease for animals and their owners [29].

## Urticaria

### Mechanisms and risk factors

Salt-dependent aquagenic urticaria is rare and has only ever been reported in adults, especially young women. Two cases of salt-dependent aquagenic urticaria have now been reported in children [30].

Two additional cases of cancer and chronic urticaria have also been reported [31, 32]. The urticaria resolved once the tumour was removed. A review of 25 previous reports of chronic urticaria and malignancy raises the possibility that CU and malignancies are linked in some patients [3].

Exposure to phthalates increases the risk of acute urticaria in children [33].

A recent study in 49 Caucasian CSU patients found elevated levels of specific IgE against a mix of *Staphylococcus aureus* enterotoxins (SEs) in 51% of patients compared to 33% in healthy controls [34]. Total serum IgE levels and CSU disease activity were correlated with *Staphylococcus enterotoxin B*-IgE (SEB-IgE) levels. These results suggest a role of SEs IgE antibodies in the pathogenesis of CSU, in keeping with the current hypothesis of autoallergy being important in some patients.

CSU patients are known to have elevated levels of C-reactive protein (CRP) and it is a sensitive inflammatory biomarker for the diagnosis and disease activity of CSU. In a retrospective study of 1253 CSU patients, higher levels of CRP were associated with autologous serum skin test positivity, arterial hypertension, urticaria activity, quality of life impairment, inflammatory and coagulation markers, and poor response to antihistamines [35].

A systematic review assessed the relationship between vitamin D and CSU [36]. Fourteen studies (1321 CSU cases and 6100 controls) were considered. Twelve studies showed statistically significant lower serum vitamin D levels in CSU patients than in the controls. Vitamin D deficiency was reported more commonly for CSU patients (34.3–89.7%) than for controls (0.0–68.9%) in 6 studies. Seven showed disease improvement after high-dosages of vitamin D supplementation. Well-designed randomized placebo-controlled studies are needed to determine the cut-off levels of vitamin D for supplementation and treatment outcomes.

### Epidemiologic studies

A physician-based online survey conducted in 5 European countries (United Kingdom, Germany, Italy, France, and Spain) assessed the annual diagnosed prevalence, disease characteristics, and treatment of CU (chronic inducible and spontaneous urticaria) and CSU in children [37]. Across the 5 European countries, the one-year diagnosed prevalence of CU and CSU in paediatric patients was 1.38% and 0.75%. This study showed a prevalence of CSU in children comparable to adults. Angioedema was reported in 6–14% of patients. A large proportion of CSU paediatric patients (40–60%) were treated with H1-antihistamines at approved doses and 16–51% received H1-antihistamines at higher doses. Approximately 1/3 of paediatric CSU patients remained uncontrolled with H1-antihistamines at approved/higher doses.

A systematic review was carried out in children under 12 years of age with CSU to assess interventions and comorbidities [38]. The systematic review included 9 reports (633 children). Five comorbidities and laboratory anomalies were found to be associated with CSU: atopy (28.1%), positive autologous serum skin test (36.8%), thyroid anomalies (6.4%) and detectable antinuclear antigen (10.4%), seroprevalence for Helicobacter pylori (21.1%), low vitamin D level (69.1%), and psychiatric disorders (70.4%). Only one study allowed for comparison with a control group.

The ASSURE-CSU (ASsessment of the Economic and Humanistic Burden of Chronic Spontaneous/Idiopathic URticaria PatiEnts) study analyzed the socio-economic burden of CSU. A recent post hoc analysis of the ASSURE-CSU study evaluated 673 patients with inadequately controlled CSU and revealed significant differences between patient- and physician-reported angioedema [39]. These were classified—according to the availability of medical records and patient-reported diagnosis—as : Yes-angioedema (concordant) (40.3%), No-angioedema (concordant) (26.9%) and Misaligned (32.8%). The frequency of angioedema in CSU patients is under-recognized by physicians, even though it has significant impact on quality-of-life, work productivity and health care resource utilization. The international EAACI/GA^2^LEN/EDF/WAO methods report for the guidelines and recommendations for the management and diagnosis of angioedema has been recently revised [40].

OPuS-2 is a Phase 3 clinical study that investigated the efficacy and safety of avoralstat, a kallikrein inhibitor, on hereditary angioedema (HAE) caused by mutations in the *SERP*-*ING1* gene that leads to a deficiency of the kallikrein inhibitor, C1 inhibitor (C1-INH) [41]. Unlike in the previous Phase 1 and Phase 2 clinical studies, the treatment efficacy with 500 mg avoralstat, 3 times daily for 12 weeks, could not be demonstrated. However, these patients experienced shortened angioedema episodes and improved QoL as assessed using the Angioedema Quality of Life Questionnaire (AE-QoL). In a separate study, the natural course of an oedematous attack in a patient with hereditary angioedema due to C1-INH deficiency was monitored for 96 h. The concentration of the C4a activation product significantly increased during the prodromal period suggesting that C4a could potentially be used as a prognostic biomarker of an edematous attack [42]. A novel type of HAE with normal C1-INH levels has been identified as having a mutation in the plasminogen gene and is manifested as swelling of the face/lips and tongue [43].

## Diagnosis and severity scores

The EAACI/GA^2^LEN/EDF/WAO guidelines for the definition, classification, diagnosis and management of urticaria have been recently revised and updated [44–46].

CSU disease activity is commonly measured using the urticaria activity scores UAS7 and UAS7_TD_. The main differences between the two is that in UAS7, symptoms are recorded daily whilst in UAS7_TD_, symptoms are recorded twice a day, and that they use different wheal scoring systems. The two different versions showed similar results when assessing the severity of 130 CSU patients, suggesting the preferential use of the simpler UAS7 scoring system [47].

## Multimorbidities

There are limited reports on the association between CU and systemic lupus erythematosus (SLE). The logistic regression analysis of 2000–2011 claims data from the Taiwanese National Health Insurance Research Database of 2105 children suffering from SLE. It is indicated that there is an increased risk of developing acute urticaria and CU, particularly in female patients [48]. A comprehensive literature review indicated that chronic hepatitis B and C are not associated with CSU and so routine screening for these viral infections in CSU patients is not necessary [49].

## Treatment

Bilastine is a H_1_-antihistamine prescribed for the treatment of CSU at a standard daily dose of 20 mg. Some patients may benefit from updosing to 40 mg and up to 80 mg for the most severe cases [50]. The X-ACT study is a clinical phase III to examine the effectiveness of omalizumab for the treatment of CSU patients with angioedema refractory to high doses of H_1_-antihistamines. The reduction in angioedema symptoms when CSU patients were treated with 300 mg omalizumab significantly improved the QoL and psychological well-being as assessed by the Angioedema Quality of Life and the Dermatology Life Quality Index (DLQI) questionnaires [51].

Findings from the Icatibant Outcome Survey, a cohort observational study, showed that the effectiveness of Icatibant for the treatment of hereditary angioedema attacks is not affected by body weight [52].

Two case reports of omalizumab being effective in normo-complementaemic urticarial vasculitis (UV) reopens discussion about the pathogenesis of UV and its relationship with CSU [53]. CSU patients have elevated levels of IgE to tissue factor and thyroglobulin which are reduced in patients treated with CSU [54]. The IgE levels can be used as a prognostic marker for the therapeutic response of omalizumab. The IgE levels in CSU patients treated with omalizumab at baseline [55] and after 4 weeks of treatment [56] were significantly lower in non-responders compared to partial and complete responders.

Even though the administration of 300 mg omalizumab may be successful in the treatment of CSU patients who do not respond to antihistamines, it does not cure the disease and patients often relapse after the regimen is completed. The high cost of the drug has prompted The Italian Medical Agency to prohibit the administration of omalizumab beyond 1 year of treatment and so there is an urgent need for alternative therapies after 1 year. A study of 14 patients with complete response to omalizumab after 6 months (300 mg/month) demonstrated that half of the patients could be switched to a regimen of 150 mg/month for an additional 4 months as an add-on treatment to second-generation antihistamines [57].

Serum sickness-like reaction was observed in a child using omalizumab for CSU [58], having previously been reported only in an adult. This is included as a warning in the summary of product characteristics.

## Conclusion

Many important papers have been published in EAACI journals this year.

## Data Availability

Not applicable.

## Abbreviations

AD: atopic dermatitis
CSU: chronic spontaneous urticaria
CU: chronic urticaria
