# Correction to: Highlights and recent developments in skin allergy and related diseases in EAACI journals (2018)

**DOI:** 10.1186/s13601-020-00344-9

**Published:** 2020-09-15

**Authors:** C. A. Akdis, J. Bousquet, C. E. Grattan, P. A. Eigenmann, K. Hofmann-Sommergruber, I. Agache, M. Jutel

**Affiliations:** 1 Swiss Institute of Allergy and Asthma Research (SIAF), University Zurich, Davos, Switzerland; 2 MACVIA-France, Fondation Partenariale FMC VIA-LR, CHU Arnaud de Villeneuve, 371 Avenue du Doyen Gaston Giraud, 34295 Montpellier Cedex 5, France; 3 INSERM U 1168, VIMA: Ageing and Chronic Diseases Epidemiological and Public Health Approaches, Villejuif Université Versailles StQuentin-en-Yvelines, UMR-S 1168, Montigny Le Bretonneux, France; 4 Charité Universitätsmedizin Berlin, Humboldt-Universität zu, Berlin, Germany; 5 Department of Dermatology and Allergy, Comprehensive Allergy Center, Berlin Institute of Health, Berlin, Germany; 6 St John’s Institute of Dermatology, Guy’s Hospital, London, UK; 7 Pediatric Allergy Unit, University Hospitals of Geneva, Geneva, Switzerland; 8 Department of Pathophysiology and Allergy Research, Medical University of Vienna, Vienna, Austria; 9 Transylvania University Brasov, Brasov, Romania; 10 Department of Clinical Immunology, Wroclaw Medical University, Wroclaw, Poland; 11 ALL-MED Medical Research Institute, Wroclaw, Poland

## Correction to: Clin Transl Allergy (2019) 9:60 10.1186/s13601-019-0299-y

Following publication of the original article [1], the authors identified an error in the affiliation list. The first affiliation for M. Jutel was omitted. This affiliation has been included as affiliation number 10:

Department of Clinical Immunology, Wroclaw Medical University, Wroclaw, Poland.

The corrected affiliation list is reflected in this Correction.
